# Association Between Iron Metabolism and Microalbuminuria in Patients With Type 2 Diabetes Mellitus and MASLD

**DOI:** 10.1155/ije/8147661

**Published:** 2026-08-29

**Authors:** Huanjia Qu, Lingling Zhou, Jinhua Hu, Dong Tang, Lu Yang

**Affiliations:** ^1^ Department of Endocrinology, the Affiliated Hospital of Hangzhou Normal University, Hangzhou 310000, China, hznu.edu.cn; ^2^ Department of Endocrinology, the Affiliated Yueqing Hospital of Wenzhou Medical University, Wenzhou 325600, China; ^3^ Department of Radiology, the Affiliated Hospital of Hangzhou Normal University, Hangzhou 310000, China, hznu.edu.cn

**Keywords:** hepatic iron content, iron metabolism, metabolic dysfunction-associated steatotic liver disease, microalbuminuria, Type 2 diabetes mellitus

## Abstract

**Objective:**

To investigate the association between iron metabolism markers and microalbuminuria in patients with Type 2 diabetes mellitus (T2DM) and metabolic dysfunction‐associated steatotic liver disease (MASLD).

**Methods:**

A total of 137 patients with T2DM and MASLD were enrolled and stratified into microalbuminuria‐positive and microalbuminuria‐negative groups based on the urinary albumin‐to‐creatinine ratio (UACR). Iron metabolism markers, including serum ferritin, serum iron, total iron‐binding capacity (TIBC), transferrin saturation (TS), and hepcidin, were measured. Hepatic iron and fat content were assessed using magnetic resonance imaging (MRI), including R2^∗^ mapping (MRI‐R2^∗^) and proton density fat fraction (MRI‐PDFF).

**Results:**

Patients with microalbuminuria had significantly higher levels of serum ferritin, serum iron, hepcidin, and MRI‐R2^∗^ compared with those without microalbuminuria (all *p* < 0.05). Correlation analyses showed that hepcidin (*r*_*s*_ = 0.208, *p* = 0.015) and MRI‐R2^∗^ (*r*_*s*_ = 0.248, *p* = 0.003) were positively associated with UACR. However, after multivariable adjustment for confounding factors, MRI‐R2^∗^ remained independently associated with microalbuminuria (*p* = 0.049).

**Conclusions:**

Altered iron metabolism is associated with microalbuminuria in patients with T2DM and MASLD. MRI‐R2^∗^, a quantitative imaging marker of hepatic iron content, showed an independent association with microalbuminuria after multivariable adjustment and may provide complementary information for early renal injury assessment in this population.

## 1. Introduction

Diabetes mellitus has emerged as a global health crisis of epidemic proportions. Current epidemiological data project that by 2045; approximately 783 million individuals worldwide will be affected by Type 2 diabetes mellitus (T2DM), with China accounting for 174 million cases [1]. This metabolic disorder poses significant health burdens primarily through its systemic complications, particularly diabetic kidney disease (DKD), which manifests initially through microalbuminuria—a sensitive marker of early renal impairment [2].

The clinical complexity of T2DM is further compounded by its frequent association with metabolic dysfunction‐associated steatotic liver disease (MASLD), the most prevalent chronic liver disease globally. In China, MASLD affects approximately 29.2% of the population and is strongly associated with obesity, metabolic syndrome, and extrahepatic complications, including chronic kidney disease (CKD) and cardiovascular disease [3]. The coexistence of T2DM and MASLD creates a vicious cycle that exacerbates systemic insulin resistance, oxidative stress, and inflammatory responses, ultimately accelerating multiorgan damage [4]. Notably, T2DM serves as both a leading cause of CKD and a shared metabolic risk factor for MASLD and CKD development [5, 6], with clinical evidence demonstrating that MASLD severity correlates with CKD progression in patients with T2DM [7, 8].

Recent advances have identified dysregulated iron homeostasis as a potential shared mechanism linking T2DM, MASLD, and renal injury [9, 10], yet this interplay remains underexplored. While iron is essential for physiological processes such as oxygen transport and cellular metabolism [11], its dysregulation contributes to disease pathogenesis through multiple mechanisms. In T2DM, iron overload promotes oxidative damage via Fenton reactions, generating reactive oxygen species (ROS) that impair insulin signaling and mitochondrial function [12]. Similarly, in MASLD, hepatic iron accumulation correlates with disease progression, driving fibrogenesis and steatohepatitis development [13]. The renal implications of iron dysregulation are increasingly recognized. Microalbuminuria, the clinical hallmark of early DKD, results from glomerular endothelial dysfunction and podocyte injury—processes that may be exacerbated by iron‐mediated oxidative stress and profibrotic signaling. Preclinical studies demonstrate that iron overload accelerates kidney injury in diabetic models, while iron restriction or chelation attenuates DKD progression. Clinical observations further support these findings, showing increased renal iron deposition in DKD patients and significant correlations between serum ferritin levels and urinary albumin excretion [14–16]. Collectively, these findings highlight the need to further investigate the role of iron metabolism in renal injury among patients with T2DM and MASLD.

Accurate assessment of iron status is critical for understanding these relationships. Among available techniques, magnetic resonance imaging (MRI)‐based proton density fat fraction (MRI‐PDFF) and R2^∗^ mapping (MRI‐R2^∗^) have emerged as reference standards for noninvasive quantification of hepatic fat and iron, respectively, offering both precision and clinical feasibility [17]. Our previous studies have demonstrated that MRI is a reliable tool for assessing liver and pancreatic fat and iron content, and we further investigated the relationship between iron metabolism and MASLD in T2DM [18, 19]. However, the specific association between iron metabolism and microalbuminuria in patients with T2DM and MASLD has not been systematically investigated. Therefore, the present study aimed to address this gap. Specifically, we investigated the associations between iron metabolism markers—including serum ferritin, serum iron, total iron‐binding capacity (TIBC), transferrin saturation (TS), hepcidin, and hepatic iron content—with microalbuminuria as assessed by the urinary albumin‐to‐creatinine ratio (UACR) in this high‐risk population.

## 2. Methods

### 2.1. Participants

This study was conducted in accordance with the Declaration of Helsinki and was approved by the Institutional Review Board of the Affiliated Hospital of Hangzhou Normal University. A total of hospitalized patients with T2DM and MASLD between June 2023 and June 2024 were enrolled. Written informed consent was obtained from all participants. Inclusion criteria were as follows: (1) a confirmed diagnosis of T2DM according to World Health Organization criteria and (2) MASLD diagnosed based on MRI‐PDFF ≥ 5.5%. Exclusion criteria were as follows: (1) age < 14 years; (2) other types of diabetes (Type 1 diabetes, gestational diabetes, or secondary diabetes); (3) significant alcohol consumption or other chronic liver diseases (e.g., viral hepatitis or drug‐induced liver disease); (4) recent major cardiovascular or cerebrovascular events (within 3 months), severe hepatic or renal dysfunction, acute infection, or severe stress conditions; (5) pregnancy or lactation; (6) disorders affecting iron metabolism (e.g., hereditary hemochromatosis or iron‐deficiency anemia), recent blood transfusion or donation, or women during menstruation. Microalbuminuria was defined as a UACR of 30–300 mg/g for study classification.

### 2.2. Data Collection

Demographic and clinical characteristics were recorded for all participants, including sex, age, height, weight, systolic blood pressure (SBP), diastolic blood pressure (DBP), medical history, comorbidities, and current medications. Body mass index (BMI) was calculated as weight (kg) divided by height (m) [2].

### 2.3. Laboratory Measurements

After a standardized 12‐h overnight fast, venous blood samples and first‐morning midstream urine specimens were collected from all participants between 6:00 and 8:00 a.m. Blood samples were collected in serum separation tubes and centrifuged at 3000 rpm for 20 min to obtain serum. Serum levels of fasting plasma glucose (FPG), alanine aminotransferase (ALT), gamma‐glutamyl transferase (GGT), alkaline phosphatase (ALP), creatinine (Cr), blood urea nitrogen (BUN), serum uric acid (SUA), serum iron, TIBC, total cholesterol (TC), low‐density lipoprotein cholesterol (LDL‐C), high‐density lipoprotein cholesterol (HDL‐C), and triglycerides (TG) were measured using an AU5800 automatic biochemistry analyzer (Beckman Coulter, Brea, CA, USA). Glycated hemoglobin (HbA1c) was measured using a Tosoh G8 HPLC automated glycohemoglobin analyzer (Tosoh Bioscience, Tokyo, Japan). Serum ferritin levels were measured using a fully automated chemiluminescence immunoassay analyzer (ARCHITECT i2000SR; Abbott Laboratories, Abbott Park, IL, USA). High‐sensitivity C‐reactive protein (hs‐CRP) levels were measured using an immunoturbidimetric method on an automated analyzer (Mindray, China). Serum hepcidin levels were quantified using a commercially available enzyme‐linked immunosorbent assay (ELISA) kit according to the manufacturer’s instructions. TS (%) was calculated as (serum iron/TIBC) × 100. The estimated glomerular filtration rate (eGFR) was calculated using the CKD‐EPI 2021 equation without a race coefficient [20]. Urinary microalbumin and creatinine were measured from a single first‐morning midstream urine sample, collected in the fasting state on the same day as blood sampling, and the UACR was calculated accordingly using a BA400 automatic specific protein analyzer (Biosystems, Barcelona, Spain).

### 2.4. Liver Stiffness Measurements (LSM)

LSM was performed using a FibroScan 502 device (Echosens, Paris, France) by trained operators. The M or XL probe was selected according to BMI (≥ 32 kg/m^2^ for the XL probe). Measurements were obtained from the right liver lobe through the intercostal spaces with the patient in the supine position and the right arm fully abducted. Only examinations with at least 10 valid measurements were considered reliable. Liver fibrosis stages were defined based on previously validated cut‐off values [21].

### 2.5. MRI Acquisition and Analysis

All participants underwent an MRI examination after at least 10 h of fasting. Imaging was performed using a 1.5 T scanner (Magnetom Avanto, Siemens Healthineers, Germany) with a 16‐channel phased‐array coil. A multiecho chemical shift‐encoded gradient‐recalled echo sequence was acquired during a single breath‐hold. MRI‐PDFF and R2^∗^ values were calculated using automated whole‐liver segmentation to assess hepatic steatosis and iron content, respectively. Hepatic steatosis was defined as MRI‐PDFF ≥ 5.5%. Three regions of interest (ROIs) were placed within the liver parenchyma by an experienced radiologist, avoiding major vessels, bile ducts, surrounding adipose tissue, and imaging artifacts. All analyses were performed with investigators blinded to clinical data.

### 2.6. Statistical Analysis

Statistical analyses were performed using SPSS version 29.0 (IBM Corp., Armonk, NY, USA). Normality and homogeneity of variance were assessed using the Shapiro–Wilk and Levene tests. Normally distributed variables were presented as mean ± standard deviation and were compared using independent‐samples t‐tests. Nonnormally distributed variables were expressed as median (interquartile range) and were analyzed using the Mann–Whitney U test. Categorical variables were presented as percentages and compared using the chi‐square test. Correlations between iron metabolism markers and UACR, as well as LSM, were assessed using Pearson or Spearman correlation analysis, depending on the data distribution. Separate binary logistic regression models were constructed for each iron metabolism marker to examine its association with microalbuminuria. Effect estimates were expressed as odds ratios (ORs) and 95% confidence intervals (CIs). Stepwise adjustment for potential confounders was applied using three models: Model 1 was adjusted for age and sex; Model 2 was additionally adjusted for BMI and hypertension; and Model 3 was further adjusted for HbA1c and SUA. A two‐sided *p* value < 0.05 was considered statistically significant.

## 3. Results

### 3.1. Characteristics of Participants

**FIGURE 1:**
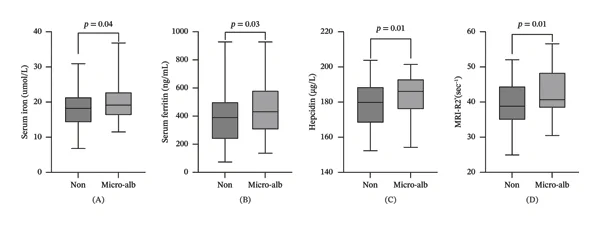
Comparison of iron metabolism markers in patients with T2DM and MASLD according to microalbuminuria status. Serum iron (A), serum ferritin (B), hepcidin (C), and MRI‐R2^∗^ (D) levels are presented. Exact *p* values are shown in the figure.

A total of 137 hospitalized patients (103 males and 34 females) were included in this study and categorized into microalbuminuria‐positive (*n* = 62) and microalbuminuria‐negative (*n* = 75) groups based on UACR levels (Table 1). No significant between‐group differences were observed in demographic characteristics (age and sex), blood pressure (SBP and DBP), prevalence of hypertension, liver enzymes (ALT and ALP), lipid parameters (TC, LDL‐C, and HDL‐C), renal function indices (Cr, eGFR, and BUN), hs‐CRP, LSM, or MRI‐PDFF (all *p* < 0.05). In contrast, patients with microalbuminuria exhibited significantly higher levels of BMI, GGT, FPG, HbA1c, TG, and SUA (all *p* < 0.05). In addition, iron metabolism markers, including serum iron (Figure 1A), serum ferritin (Figure 1B), hepcidin (Figure 1C), and MRI‐R2^∗^ (Figure 1D), were significantly higher in the microalbuminuria‐positive group (all *p* < 0.05), whereas no significant differences were observed in TIBC or TS.

**TABLE 1:** Characteristics of participants according to microalbuminuria status.

Variables	All (*n* = 137)	Microalbuminuria (−) (*n* = 75)	Microalbuminuria (+) (*n* = 62)	*p* value
Age (years)	42.00 (33.00, 51.00)	42.00 (35.00, 53.00)	38.00 (29.00, 50.00)	0.197
Sex (male/female)	103/34	55/20	48/14	0.582
BMI (kg/m2)	27.50 (24.85, 30.10)	26.80 (24.70, 29.30)	28.30 (24.90, 32.00)	0.025
SBP (mmHg)	134.51 ± 16.10	133.60 ± 16.12	135.61 ± 16.13	0.468
DBP (mmHg)	84.85 ± 11.62	83.63 ± 11.37	86.32 ± 11.85	0.177
Hypertension, *n* (%)	40 (29.18%)	20 (26.67%)	20 (32.26%)	0.474
ALT (U/L)	35.00 (23.00, 65.00)	35.00 (24.00, 71.00)	31.00 (22.00, 63.00)	0.592
GGT (U/L)	46.00 (32.00, 79.00)	39.00 (29.00, 68.00)	53.00 (36.00, 93.00)	0.008
ALP (U/L)	86.00 (71.50, 103.00)	84.00 (74.00, 98.00)	91.00 (64.00, 112.00)	0.366
FPG (mmol/L)	14.18 (11.23, 17.51)	13.38 (9.78, 16.70)	15.90 (13.39, 18.17)	0.003
HbA1c (%)	11.30 (10.00, 12.45)	11.00 (10.00, 12.10)	11.8 (10.10, 13.70)	0.013
TC (mmol/L)	5.13 (4.51, 6.14)	5.09 (4.33, 5.95)	5.16 (4.53, 6.24)	0.151
TG (mmol/L)	3.00 (1.63, 5.98)	2.32 (1.40, 4.30)	4.01 (2.19, 6.87)	0.003
LDL‐C (mmol/L)	3.27 (2.81, 3.71)	3.22 (2.52, 3.62)	3.30 (2.81, 3.92)	0.335
HDL‐C (mmol/L)	1.04 (0.91, 1.18)	1.00 (0.87, 1.18)	1.04 (0.94, 1.18)	0.263
SUA (μmol/L)	356.81 ± 130.97	329.72 ± 93.84	389.59 ± 159.89	0.010
Cr (μmol/L)	62.30 (52.70, 72.15)	63.60 (54.10, 73.30)	61.30 (51.60, 69.80)	0.352
eGFR(mL/min/1.73 m^2^)	114.08 (105.61, 125.82)	113.33 (102.88, 123.16)	118.55 (106.72, 130.31)	0.078
BUN (mmol/L)	5.19 (4.23, 5.98)	5.24 (4.55, 5.75)	4.84 (4.03, 6.25)	0.533
UACR (mg/g)	21.13 (8.84, 77.31)	9.28 (5.92, 12.73)	82.12 (43.27, 131.38)	—
hs‐CRP (mg/L)	2.12 (1.08, 6.04)	2.16 (1.17, 5.33)	1.91 (1.00, 6.55)	0.886
LSM (kPa)	6.10 (4.60, 8.40)	5.90 (5.00, 7.10)	6.4 (4.6, 8.7)	0.583
Serum ferritin (ng/mL)	423.21 ± 191.77	391.66 ± 190.50	461.36 ± 187.79	0.034
Serum iron (μmol/L)	18.75 ± 4.97	17.97 ± 4.94	19.70 ± 4.89	0.042
TIBC (μmol/L)	54.70 (50.35, 60.90)	54.00 (49.90, 60.30)	57.30 (51.10, 62.10)	0.181
TS (%)	32.84 (26.62, 39.28)	32.69 (25.06, 39.18)	33.94 (30.49, 39.80)	0.174
Hepcidin (μg/L)	181.91 (173.06, 191.26)	179.80 (168.26, 188.64)	186.09 (175.90, 193.05)	0.013
MRI‐R2^∗^ (sec^−1^)	39.71 (36.23, 45.85)	38.78 (34.90, 44.45)	40.67 (38.34, 48.37)	0.013
MRI‐PDFF (%)	12.36 (7.85, 18.50)	13.35 (7.56, 19.41)	11.30 (7.85, 17.58)	0.854

*Note:* Data are presented as mean ± standard deviation (SD), median (interquartile range, IRQ), or *n* (%). *p* values were calculated using the independent‐samples t‐test, Mann–Whitney U test, or chi‐square test, as appropriate. ALP, alkaline phosphatase; ALT, alanine aminotransferase; Cr, creatinine; HbA1c, glycated hemoglobin; TG, triglycerides; UACR, urinary albumin‐to‐creatinine ratio.

Abbreviations: BMI, body mass index; BUN, blood urea nitrogen; eGFR, estimated glomerular filtration rate; FPG, fasting plasma glucose; GGT, gamma‐glutamyl transferase; HDL‐C, high‐density lipoprotein cholesterol; hs‐CRP, high‐sensitivity C‐reactive protein; LDL‐C, low‐density lipoprotein cholesterol; LSM, liver stiffness measurement; MRI‐PDFF, magnetic resonance imaging proton density fat fraction; MRI‐R2^∗^, magnetic resonance imaging R2^∗^; SUA, serum uric acid; TC, total cholesterol; TIBC, total iron‐binding capacity; TS, transferrin saturation.

### 3.2. Correlations Between UACR and Iron Metabolism Markers

**FIGURE 2:**
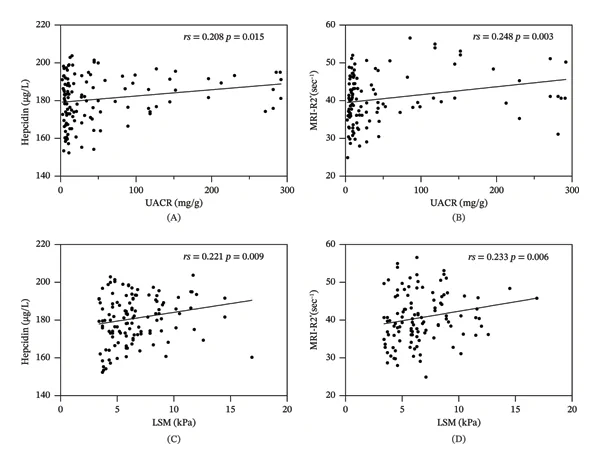
Correlations of UACR and LSM with iron metabolism markers. UACR was positively correlated with hepcidin (A) and MRI‐R2^∗^ (B), while LSM was positively correlated with hepcidin (C) and MRI‐R2^∗^ (D). Correlation coefficients and exact *p* values are shown in the figure.

UACR was positively correlated with hepcidin (*r*_*s*_ = 0.208, *p* = 0.015) and MRI‐R2^∗^ (*r*_*s*_ = 0.248, *p* = 0.003). No significant correlations were observed between UACR and serum ferritin (*r* = 0.143, *p* = 0.095), serum iron (*r* = 0.100, *p* = 0.246), TIBC (*r*_*s*_ = 0.119, *p* = 0.167), or TS (*r*_*s*_ = 0.050, *p* = 0.565) (Table 2; Figure 2A,B).

**TABLE 2:** Correlations between UACR and iron metabolism markers.

Variables	Correlation coefficient (*r*/*r*_*s*_)	*p* value
Serum ferritin (ng/mL)	*r* = 0.143	0.095
Serum iron (μmol/L)	*r* = 0.100	0.246
TIBC (μmol/L)	*r*_*s*_ = 0.119	0.167
TS (%)	*r*_*s*_ = 0.050	0.565
Hepcidin (μg/L)	*r*_*s*_ = 0.208	0.015
MRI‐R2^∗^ (sec^−1^)	*r*_*s*_ = 0.248	0.003

*Note:* Correlations were analyzed using Pearson (*r*) or Spearman (*r*_*s*_) correlation analysis, as appropriate.

Abbreviations: MRI‐R2^∗^, magnetic resonance imaging R2^∗^; TIBC, total iron‐binding capacity; TS, transferrin saturation; UACR, urinary albumin‐to‐creatinine ratio.

### 3.3. Correlations Between LSM and Iron Metabolism Markers

LSM was positively correlated with hepcidin (*r*_*s*_ = 0.221, *p* = 0.009) and MRI‐R2^∗^ (*r*_*s*_ = 0.233, *p* = 0.006). No significant correlations were observed between LSM and serum ferritin (*r*_*s*_ = −0.012, *p* = 0.893), serum iron (*r*_*s*_ = −0.003, *p* = 0.970), TIBC (*r*_*s*_ = 0.088, *p* = 0.306), or TS (*r*_*s*_ = −0.075, *p* = 0.383) (Table 3; Figure 2C,D).

**TABLE 3:** Correlations between LSM and iron metabolism markers.

Variables	Correlation coefficient (*r*_*s*_)	*p* value
Serum ferritin (ng/mL)	*r*_*s*_ = −0.012	0.893
Serum iron (μmol/L)	*r*_*s*_ = −0.003	0.970
TIBC (μmol/L)	*r*_*s*_ = 0.088	0.306
TS (%)	*r*_*s*_ = −0.075	0.383
Hepcidin (μg/L)	*r*_*s*_ = 0.221	0.009
MRI‐R2^∗^ (sec^−1^)	*r*_*s*_ = 0.233	0.006

*Note:* Correlations were analyzed using Spearman (*r*_*s*_) correlation analysis.

Abbreviations: LSM, liver stiffness measurement; TIBC, total iron‐binding capacity; TS, transferrin saturation; MRI‐R2^∗^, magnetic resonance imaging R2^∗^.

### 3.4. Associations Between Iron Metabolism Markers and Microalbuminuria in Patients With T2DM and MASLD

Binary logistic regression analysis was performed to evaluate the association between iron metabolism markers and microalbuminuria (Table 4). In Model 1 (adjusted for age and sex), higher levels of MRI‐R2^∗^, serum ferritin, serum iron, and hepcidin were significantly associated with microalbuminuria (all *p* < 0.05). After additionally adjusting for BMI and hypertension (Model 2), MRI‐R2^∗^ and hepcidin remained significantly associated with microalbuminuria (both *p* < 0.05). In the fully adjusted model (Model 3), only MRI‐R2^∗^ remained independently associated with microalbuminuria (*p* = 0.049), whereas hepcidin did not reach statistical significance (*p* = 0.052).

**TABLE 4:** Associations between iron metabolism markers and microalbuminuria in patients with T2DM and MASLD.

Variables	Model 1		Model 2		Model 3	
	OR (95% CI)	*p* value	OR (95% CI)	*p* value	OR (95% CI)	*p* value
Serum ferritin (ng/mL)	1.002 (1.000–1.004)	0.045	1.002 (1.000–1.004)	0.063	1.002 (1.000–1.004)	0.073
Serum iron (μmol/L)	1.078 (1.002–1.159)	0.044	1.072 (0.995–1.155)	0.066	1.080 (0.998–1.169)	0.056
Hepcidin (μg/L)	1.038 (1.007–1.069)	0.017	1.034 (1.002–1.066)	0.035	1.033 (1.000–1.067)	0.052
MRI‐R2^∗^ (sec^−1^)	1.082 (1.022–1.145)	0.007	1.071 (1.010–1.136)	0.022	1.063 (1.000–1.130)	0.049

*Note:* Separate binary logistic regression models were constructed for each iron metabolism marker. ORs represent the risk of microalbuminuria per unit increase in each variable. T2DM, Type 2 diabetes mellitus; MASLD, metabolic dysfunction‐associated steatotic liver disease; HbA1c, glycated hemoglobin. Model 1: adjusted for age and sex. Model 2: additionally adjusted for BMI and hypertension. Model 3: further adjusted for HbA1c and SUA.

Abbreviations: CI, confidence interval; OR, odds ratio; SUA, serum uric acid.

## 4. Discussion

Our study suggests that altered iron metabolism is associated with early renal injury in patients with T2DM and MASLD. Notably, MRI‐R2^∗^, a quantitative imaging marker of hepatic iron content, showed an independent association with microalbuminuria after adjustment for potential confounders. These findings highlight the potential role of MRI‐based hepatic iron assessment as a complementary approach for evaluating early renal injury in this population.

Our findings are consistent with previous reports linking iron overload to the pathogenesis of DKD [22]. However, by integrating serological and imaging‐based iron markers, we further delineate the relative contributions of different iron metabolism markers.

In initial analyses, multiple iron‐related markers, including serum iron, ferritin, hepcidin, and MRI‐R2^∗^, were significantly elevated in patients with microalbuminuria. Correlation analyses further identified significant associations of hepcidin and MRI‐R2^∗^ with UACR. Nevertheless, these associations were progressively attenuated after adjustment for confounders, with only MRI‐R2^∗^ remaining independently associated with microalbuminuria in the fully adjusted model. The loss of significance for serum iron and ferritin is biologically plausible and consistent with their inherent limitations. Ferritin, as an acute‐phase reactant, is frequently elevated in chronic low‐grade inflammatory states such as T2DM and MASLD, while serum iron is subject to short‐term fluctuations influenced by dietary intake and circadian variation [23]. Similarly, although hepcidin was elevated and initially associated with UACR, it was no longer independently associated with microalbuminuria in the fully adjusted model. This may reflect its complex regulation by metabolic and inflammatory factors, which are highly prevalent in T2DM and MASLD [24–26]. In contrast, MRI‐R2^∗^, as an imaging‐based measure of hepatic iron content, may better reflect tissue‐level iron deposition. The independent association observed for MRI‐R2^∗^ in our study indicates that tissue iron accumulation, rather than circulating iron markers, may serve as a more stable and clinically relevant indicator of early renal injury. These findings further support the concept of a liver–kidney axis in iron‐mediated organ damage, whereby MASLD‐related hepatic iron overload may contribute to renal injury through systemic oxidative stress and metabolic dysregulation [9]. Additionally, the observed correlations between LSM and both hepcidin and MRI‐R2^∗^ suggest that iron accumulation may be linked not only to renal injury but also to hepatic fibrotic burden, supporting a shared pathophysiological pathway across the liver–kidney axis.

The observed associations may be explained by several interconnected mechanisms. First, excess iron catalyzes Fenton reactions, generating ROS that directly damage glomerular endothelial cells and podocytes, thereby increasing albumin permeability [27]. Second, elevated hepcidin—by binding to and inducing internalization of ferroportin—reduces cellular iron export, leading to iron sequestration within renal tubular cells and macrophages. This retained iron perpetuates local oxidative injury through sustained ROS production [28]. Third, hepatic steatosis promotes the release of proinflammatory cytokines, which further stimulate hepcidin production and exacerbate systemic iron redistribution from storage sites to the kidneys [29]. Fourth, iron overload impairs insulin signaling in glomerular endothelial cells, reducing nitric oxide bioavailability and exacerbating glomerular hyperfiltration—an early hemodynamic abnormality in DKD pathogenesis [30]. Overall, these mechanisms suggest a vicious cycle among T2DM, MASLD, and iron metabolic disorders that accelerates early kidney injury, with tissue‐level iron deposition potentially playing a central role.

From a clinical perspective, MRI‐based hepatic iron quantification may serve as a noninvasive tool to identify patients at increased risk of early kidney injury. Compared with conventional iron biomarkers, MRI‐R2^∗^ appears to provide greater stability and independence from metabolic confounders. Patients with iron metabolism disorders, especially hepatic iron deposition, may benefit from closer monitoring of renal function, particularly through regular assessment of UACR. However, whether targeting iron metabolism improves renal outcomes in this population remains to be determined.

Several limitations should be acknowledged. The cross‐sectional design precludes the determination of causal relationships between iron metabolism markers and the development of microalbuminuria. The low number of subjects may limit statistical power to detect weaker associations, particularly in stratified analyses. In addition, UACR was assessed using a single morning urine sample without repeated measurements to confirm persistent microalbuminuria, which may limit diagnostic accuracy. The absence of histopathological data represents another constraint, as does the potential influence of unmeasured confounders, including genetic polymorphisms and dietary iron intake patterns. Furthermore, as a single‐center investigation using 1.5 T MRI, our findings require validation across different imaging platforms and healthcare settings. Future studies should focus on (1) multicenter longitudinal studies to establish temporal relationships between iron homeostasis and DKD progression; (2) mechanistic investigations using animal models to elucidate renal iron transport pathways; (3) clinical trials evaluating iron reduction therapies in patients with T2DM and MASLD; and (4) genetic analyses to identify high‐risk subgroups based on iron regulatory gene variants. These approaches would help translate our observational findings into clinically actionable knowledge [31].

## 5. Conclusion

In conclusion, this study suggests that altered iron metabolism, particularly elevated hepatic iron content, is associated with microalbuminuria in patients with T2DM and MASLD. Among the evaluated iron‐related indicators, MRI‐R2^∗^ showed an independent association with early renal injury after adjustment for potential confounders. These findings suggest a potential link between hepatic iron accumulation and renal injury and indicate that imaging‐based iron assessment may provide complementary information for early renal injury assessment. Given the exploratory nature of these findings, further longitudinal and mechanistic studies are warranted to clarify causality and determine whether targeting iron metabolism may offer therapeutic benefits.

## Author Contributions

All authors took part in drafting, revising or critically reviewing the article.

## Funding

This research was supported by the Medical Science and Technology Project of Zhejiang Province (2023RC234) and Hangzhou Health Science and Technology Programme (A20220027).

## Disclosure

All authors gave final approval of the version to be published; have agreed on the journal to which the article has been submitted; and agreed to be accountable for all aspects of the work.

## Ethics Statement

The study was approved by the Ethics Committee of the Affiliated Hospital of Hangzhou Normal University (No.2022‐(E2)‐HS‐146) and conducted by the Declaration of Helsinki.

## Consent

All participants provided written informed consent.

## Conflicts of Interest

The authors declare no conflicts of interest.

## Data Availability

The original contributions of this study are contained within the article and the data supporting the findings are available upon reasonable request. For further inquiries, please contact the corresponding author.
